# Malnutrition-Inflammation Complex Syndrome: A Cause of Low Parathyroid Hormone in Patients With Chronic Kidney Disease

**DOI:** 10.7759/cureus.20324

**Published:** 2021-12-10

**Authors:** Rishi Raj, Aditya Kadiyala, Chinmay Patel

**Affiliations:** 1 Endocrinology, Diabetes and Metabolism, Pikeville Medical Center, Pikeville, USA; 2 Nephrology, University of Maryland Charles Regional Medical Center, La Plata, USA; 3 Nephrology, East Texas Kidney Specialists, Longview, USA

**Keywords:** malnutrition inflammation complex syndrome, inflammation, protein energy malnutrition, chronic kidney disease (ckd), parathyroid hormone

## Abstract

Secondary hyperparathyroidism is commonly seen in patients with chronic kidney disease (CKD) due to hypocalcemia, hyperphosphatemia and low vitamin D levels and is associated with high-turnover bone disease. In contrast, some patients with advanced CKD, including those requiring dialysis (end-stage renal disease [ESRD]), develop adynamic bone disease with features of low-turnover bone disease. Low serum parathyroid hormone (PTH) has been used as a biochemical marker of adynamic bone disease. Low PTH levels may not necessarily be due to adynamic bone disease but could be a manifestation of the malnutrition inflammation complex syndrome (MICS). The optimal management of hypoparathyroidism associated with MICS is not well known. Currently, there is insufficient evidence to suggest if there is any role in improving nutritional and inflammatory status among patients with CKD and MICS. Furthermore, it also remains unclear whether these changes will help address low PTH levels seen in these patients. We report three patients with advanced CKD who had very low PTH levels possibly attributed to MICS. In addition, we briefly discuss other characteristics and pathophysiology of MICS.

## Introduction

Secondary hyperparathyroidism (serum PTH > 65 pg/mL) is common in patients with chronic kidney disease (CKD), and with decreasing estimated glomerular filtration rate (EGFR) levels, the majority of the patients (>80%) have PTH > 150 pg/mL [1]. Secondary hyperparathyroidism is associated with vascular calcification, increased bone turnover disease, greater fracture rates, as well as higher mortality. Adynamic bone disease (ABD) is a type of CKD-osteodystrophy commonly induced by overtreatment of secondary hyperparathyroidism, and its development reveals a deranged ability of uremic bone to maintain a normal bone turnover [1]. The term MICS has been proposed to indicate the combination of malnutrition and inflammation, which tend to coexist and occur concomitantly in patients with end-stage renal disease (ESRD) [2]. Low serum PTH is seen with protein-energy malnutrition and is also considered a marker of inflammation. Hence, it may not necessarily represent ABD [2]. While withholding PTH suppressive medications has been suggested in patients with ESRD with PTH levels below 150 pg/mL, optimal management of ESRD patients with low PTH, and MICS, who are not on any PTH suppressive therapy remains unclear.

## Case presentation

Case 1

A 47-year-old white female with a medical history of type 2 diabetes mellitus, coronary artery disease, nonischemic cardiomyopathy, hypertension, hyperlipidemia, left foot osteomyelitis, chronic diabetic foot ulcer, peripheral artery disease, CKD stage 4, chronic pain syndrome, and chronic obstructive pulmonary disease was first evaluated by nephrology in May 2018 for CKD stage 3b. On initial evaluation, serum creatinine was 1.7 mg/dL with an EGFR of 32.4 mL/min/1.73m^2^. CKD was attributed to underlying diabetic nephropathy. Additional laboratory workup showed a low PTH of 9.3 pg/mL with normal calcium of 9.5 mg/dL, phosphorus level of 4 mg/dL, and 25 hydroxy Vitamin D levels 31 ng/mL. The rest of the laboratory workup is summarized in Table 1. There was no history of thyroid surgery or radiation to the neck. Repeat biochemical workup at one year and two years' follow-up visits revealed persistently suppressed PTH levels along with normal calcium levels and phosphorus levels, despite progression of CKD. Between 2018 and 2020, she had multiple hospitalizations secondary to infection of diabetic foot ulcer, pulmonary infection, and volume overloads secondary CKD and cardiomyopathy.

**Table 1:** Laboratory workup of patient 1

Laboratory test	Reference range	05/2018	04/2019	03/2020
Hemoglobin	11-17 mg/dL	10.1	10.2	11.5
Hematocrit	32-51.6%	31	31.5	33.6
White blood cells	3-11.3 K/µL	13.6	13.1	13.4
Platelets	134-412 K/µL	304	247	245
Serum glucose	70-110 mg/dL	116	165	56
Serum sodium	133-144 mmol/L	138	138	137
Serum potassium	3.6-5.2 mmol/L	4	4.4	4.1
Serum calcium	8.3-10.4 mg/dL	9.5	9.9	9.7
Serum magnesium	1.7-2.4 mg/dL	2.1	2.1	2.1
Serum bicarbonate	21-32 mmol/L	23	27	28
Serum phosphorus	3.5-5.0 mg/dL	4	4.8	4.6
Serum uric acid	3.5-7.2 mg/dL	NA	NA	NA
Blood urea nitrogen	6-24 ng/dL	29	47	52
Serum creatinine	0.60-1.10 mg/dL	1.7	2.6	3.2
Estimated glomerular filtration rate	(>60 mL/min/1.73m^2^)	32.4	19.8	18.1
Serum albumin	3.4-5.4 mg/dL	3.1	2.9	3.2
Serum ionized calcium	1.19-1.29 mmol/L	NA	1.21	1.07
Serum aspartate transaminases	15-37 U/L	NA	8	45
Serum alanine transaminases	12-78 U/L	NA	18	52
Serum alkaline phosphatase	44-147 U/L	NA	184	172
PTH, intact	8.0-74.0 pg/mL	9.3	< 6.3	<6.3
25 OH vitamin D	20-80 ng/mL	31	20.9	25.6
1,25-OH vitamin D	20 to 45 pg/mL	19.9	NA	NA
Hemoglobin A1C	<6.5%	7.1	9	9.6
Urine microalbumin, Random	1.3-30 mg/dL	74	112	NA
Erythrocyte sedimentation rate	(0-20 mm/hr)	NA	98	88
C-reactive protein	(0-0.3 mg/dL)	NA	4.2	2
Urine protein creatinine ratio	<0.2	0.7	0.62	1.02

Case 2

An 81-year-old white male with a medical history of type II diabetes mellitus, peripheral neuropathy, hypertension, hyperlipidemia, peripheral vascular disease, and CKD was first evaluated by the Nephrology team in October 2019 for CKD stage 3b. At that time, laboratory workup revealed low PTH 33.5 pg/mL, with calcium 9.4 mg/dL, serum creatinine 1.7 mg/dL, BUN 48 mg/dL, and EGFR 38.9 mL/min/1.73m^2^. The rest of the laboratory workup is summarized in Table 2. In January 2020, serum creatinine increased to 2.2 mg/dL; calcium levels remained normal at 9.2 mg/dL, and phosphorus was high normal 4.7 mg/dL. In March 2020, he was hospitalized for worsening right ankle ulcer and osteomyelitis of the right fibula. He had a complicated hospital course and developed healthcare-associated pneumonia (HCAP) requiring management in the intensive care unit (ICU). Following discharge, he was hospitalized multiple times (urinary tract infections, pneumonia, and respiratory failure) between April and May 2020. Due to recurrent hospitalizations, he developed worsening debility and malnutrition, required feeding tube placement for nutrition management. Eventually, he developed a sacral decubitus ulcer and multiple other pressure ulcers on heels and ankles, requiring regular wound care prolonged antibiotic therapy. Subsequently, he developed ESRD and was initiated on renal replacement therapy in August 2020. A standard dialysis bath using dialysate calcium concentration of 2.5 mEq/L was used during dialysis.

**Table 2:** Laboratory workup of patient 2

Laboratory Test	Reference range	10/2019	01/2020	08/2020	11/2020
Hemoglobin	11-17 mg/dL	11.7	NA	8.4	6.6
Hematocrit	32-51.6%	35.5	NA	26.6	22
White blood cells	3.5-13 K/µL	7.2	NA	14.3	6.9
Platelet	134-412 K/µL	221	NA	405	367
Serum glucose	70-110 mg/dL	147	46	238	164
Serum sodium	133-144 mmol/L	137	139	NA	NA
Serum potassium	3.6-5.2 mmol/L	5.3	4.7	NA	NA
Serum calcium	8.3-10.4 mg/dL	9.4	9.4	9.4	7.6
Serum magnesium	1.7-2.4 mg/dL	NA	NA	NA	NA
Serum bicarbonate	21-32 mmol/L	NA	NA	22	30
Serum phosphorus	3.5-5.0 mg/dL	3.5	4.3	3.6	5
Serum uric acid	3.5-7.2 mg/dL	8	NA	NA	NA
Blood urea nitrogen	6-24 ng/dL	48	62	16	14
Serum creatinine	0.60-1.10 mg/dL	1.70	2.20	2.4	2.18
Estimated glomerular filtration rate	>60 mL/min/1.73m^2^	38.9	28.9	NA	NA
Serum albumin	3.4-5.4 mg/dL	3.4	3.7	2.7	2
Serum aspartate transaminases	8-48 U/L	NA	NA	18	12
Serum alanine transaminases	7-45 U/L	NA	NA	11	<9
Serum alkaline phosphatase	44-147 IU/L	NA	NA	123	68
PTH, intact	8.0-74.0 pg/mL	33.5	NA	10	23
25-OH vitamin D	20-80 ng/mL	27.8	NA	18.3	26.3
1,25-OH vitamin D	20 to 45 pg/mL	NA	NA	NA	NA
Hemoglobin A1C	<6.5%	NA	NA	6.3	6
Urine microalbumin, Random	1.3-30 mg/dL	57.5	NA	NA	NA

Case 3

A 51-year-old white female with a medical history of type 2 diabetes mellitus, obesity, hypertension, and low back pain was first evaluated by Nephrology in March 2015 for CKD stage 3a. She was not a smoker and had no family history of kidney disease. At the initial visit, the patient was found to have a serum creatinine of 1.38 with an EGFR of 51 mL/min/1.73m^2^. The patient initially had subnephrotic range proteinuria of 1,000 mg/g, which was attributed to early diabetic nephropathy. Her other workup for secondary causes of CKD was unremarkable. Initial bone mineral laboratory studies in March 2015 showed PTH 99 pg/mL and 25-OH vitamin D 40 ng/mL on a maintenance dose of vitamin D3 5,000 IU daily. CKD remained stable for two years until March 2017, when she was hospitalized for community-acquired pneumonia. Following this hospitalization, her glycemic control deteriorated. In December 2017, her creatinine worsened to 1.79 mg/dL with an EGFR of 32 mL/min/1.73m^2^. At that time, her PTH was 39 pg/mL and her 25-OH vitamin D was 34 ng/mL. She was taken off all vitamin D supplements. Her clinical course deteriorated further, wherein she suffered multiple hospitalizations over the next two years for fluid overload, transient ischemic attack, and a cerebrovascular accident. This happened in the setting of poorly controlled diabetes, hypertension associated with nephrotic range proteinuria, and progressive CKD. Surprisingly, as her creatinine and EGFR worsened, her PTH levels started trending down and remained suppressed despite low vitamin D levels. The trend of her laboratory workup is summarized in Table 3.

**Table 3:** Laboratory workup of patient 3

Laboratory test	Reference range	03/2015	12/2017	06/2018	06/2019	11/2020
Hemoglobin	11-17 mg/dL	11.5	12.3	12.3	11.7	12.1
Serum glucose	70-110 mg/dL	181	91	116	173	164
Serum calcium	8.3-10.4 mg/dL	9.3	9.4	NA	10.3	10.6
Serum phosphorus	3.5-5.0 mg/dL	3.2	4.1	5.2	4.9	5.0
Serum creatinine	0.60-1.10 mg/dL	1.38	1.79	2.2	2.95	3.9
Estimated glomerular filtration rate	>60 mL/min/1.73m^2^	51	32	29	17	12
Serum albumin	3.4-5.4 mg/dL	3.8	3.7	4.0	3.3	2.8
Serum alkaline phosphatase	44-147 U/L	73	NA	133	108	146
PTH, intact	8.0-74.0 pg/mL	99	39	NA	7	6.8
25-OH vitamin D	20-80 ng/mL	40	34	NA	16	21
Hemoglobin A1C	<6.5%	9.2	NA	10.2	8.2	7.0
Urine protein creatinine ratio	<0.2	1	0.947	NA	10.078	NA

## Discussion

PTH levels tend to rise with CKD progression especially with a decline in EGFR below 45 mL/min/1.73m^2^. Patients with advanced CKD, including patients with ESRD on renal replacement therapy, eventually develop CKD-mineral bone disorder (MBD) [1]. As the kidney function declines, calcium and phosphate levels are initially kept within normal levels by compensatory mechanisms. Eventually, there are elevations in fibroblast growth factor 23 (FGF-23) levels, which is considered to be the first biochemical abnormality. It decreases 1-alpha hydroxylase activity in the kidneys, decreasing the conversion of 25-hydroxy vitamin D to 1,25 dihydroxy vitamin D, and stimulates 24-hydroxylase activity leading to increased vitamin D degradation. In the setting of 1,25 dihydroxy vitamin D deficiency and hypocalcemia, parathyroid cells proliferate and ramp up the PTH synthesis. Hyperphosphatemia, which is commonly seen among patients with advanced CKD, can further stimulate PTH production. Elevated PTH then stimulated 1-alpha hydroxylase expressions thereby mobilizing calcium from bones. Hence, secondary hyperparathyroidism is considered both a cause and effect of bone mineral disorder. Furthermore, it has also been associated with CKD osteodystrophy with high bone turnover, greater fracture risk, poor quality of life, and high mortality [1]. Biomarkers, such as PTH and bone alkaline phosphatase, are only modestly predictive of underlying bone histology but currently are the best available noninvasive tools for the assessment of CKD osteodystrophy [3].

ABD is a type of CKD osteodystrophy characterized by reduced osteoblastic activity, osteoclastic activity, and markedly low bone turnover. It is more commonly seen in patients on peritoneal dialysis, including those with diabetes, advanced age, and the non-black race [3]. ABD is usually associated with a low PTH level and is commonly seen in the setting of excessive vitamin D administration, relative hypercalcemia (calcium-rich diet, calcium-based phosphate binders, higher calcium dialysate bath), administration of calcimimetics, and recombinant PTH (teriparatide), post parathyroidectomy or due to PTH assay errors [3]. Current guidelines for patients with ESRD, such as Kidney Disease Outcome Quality Initiative (KDOQI) and the Kidney Disease Improving Global Outcomes (KDIGO) 2009 suggest a target PTH range of 150 to 300 pg/mL and 130-600 pg/mL, respectively. Most recently, 2017 KDIGO guidelines for MBD recommend treating secondary hyperparathyroidism based on the individual patient’s temporal PTH trend [3]. Based on these recommendations, secondary hyperparathyroidism is treated with vitamin D, activated vitamin D, and cinacalcet while withholding PTH suppressing medications such as active vitamin D and/or calcimimetics if serum PTH is below 150 pg/mL [4].

Hyperglycemia can also impact PTH secretion through a complex interplay. While short-term hyperglycemia can result in urinary calcium loss, resulting in mild stimulation in PTH secretion, long-term hyperglycemia results in significantly attenuated PTH response [5,6]. Protein-energy malnutrition is a common phenomenon in patients with advanced CKD and ESRD and is attributed to inadequate nutrition due to anorexia and dietary restrictions, abnormal nutrient metabolism due to hyper catabolism, and increased nutrient loss during dialysis. The prevalence of protein-energy malnutrition among ESRD patients varies between 18% and 75%, depending on dialysis modality, nutritional assessment tools, and patient population [7]. Furthermore, the majority of these patients (up to two-thirds) can have cachexia along with increased levels of cytokines, suggestive of acute and/or chronic inflammation to be common among these patients, especially ESRD [4]. This is due to many underlying factors, including the uremic milieu, volume overload with endotoxemia, decreased clearance of circulating proinflammatory cytokines, oxidative and carbonyl stress, decreased levels of antioxidants, protein-energy malnutrition, the enhanced incidence of infections, and others [7]. In prolonged and persistent stages of inflammation, it leads to hypercatabolism, anorexia, muscle and/or fat wasting, endothelial damage with resultant atherosclerosis. Thus, the degree to which there is an interconnection between protein-energy malnutrition and inflammation and its independent impact in patients with kidney disease is unclear. In patients with ESRD, the markers of MICS (such as low albumin, low BMI, and/or elevated CRP) are also the predictors that are considered cardiovascular risk factors and are associated with higher mortality [8].

Low PTH has also been found to be associated with MICS in patients with CKD [4]. Low PTH leads to decreased accumulation of adipose tissue, which may ultimately result in protein-energy malnutrition states [9]. Additionally, in vitro PTH secretion has been shown to suppress pro-inflammatory cytokines associated with poor outcomes in ESRD patients, and hence a low PTH seen in MICS may be associated with increased mortality [10,11]. Among patients on chronic peritoneal dialysis, low albumin can also be seen with ABD [12]. A Japanese study on more than 15,000 patients with ESRD found an association between reduced PTH levels (<60 pg/mL) and low albumin, blood urea nitrogen levels [13]. Multiple other studies have found a direct relationship between PTH levels and creatinine, albumin, prealbumin, and cholesterol levels [14,15].

Most epidemiologic studies have suggested that the association between PTH and all-cause mortality in ESRD patients is U-shaped. A recent prospective observational study demonstrated that a low PTH level is also an independent risk factor for infection-related mortality in incident dialysis patients [16]. An observational study by Dukkipati et al. found that in ESRD patients, low serum PTH (<150 pg/mL) was associated with low serum albumin, creatinine, total iron-binding capacity (TIBC), and low percentage of lymphocytes, which are markers of protein-energy wasting and inflammation. MICS is negatively associated with low serum PTH, and this association significantly modifies the expected association between serum PTH and alkaline phosphatase in high ranges of PTH [4]. Hence, it may be speculated that MICS plays a primary role in suppressing PTH levels in the setting of normal or high turnover bone status. Few inflammatory markers (IL-6, CRP, and TNF-alpha) have often been found to be associated with the low serum PTH < 150pg/mL, often misinterpreted as ABD [2].

There is a paucity of the current consensus on MICS, in terms of the degree of severity and its management. Several scoring systems have been proposed, such as the “malnutrition-inflammation score” (http://www.touchcalc.com/calculators/mis) [17]. MIS correlates strongly with the measures of nutritional status and inflammation and is also significantly associated with hospitalization rates and mortality in ESRD patients on hemodialysis [17,18]. The Subjective Global Assessment of Nutrition (SGA) is recommended in the National Kidney Foundation KDOQI guidelines to be used routinely to assess the nutritional status of ESRD patients. The SGA also helps measure the degree of inflammation and severity of illness [19].

All the three patients that we presented showed a suppressed PTH level despite their advanced CKD while not taking any PTH suppressive medications like Vitamin D analogs or calcimimetics. Patient 1 had multiple infections causing a chronic inflammatory state, as evident by her elevated erythrocyte sedimentation rate (ESR) and C-reactive protein (CRP). Her underlying medical conditions, such as diabetes and cardiomyopathy, could have contributed to malnutrition, evident by her low albumin level. Similarly, patient 2 had osteomyelitis and later on developed chronic respiratory failure leading to debility and malnutrition requiring placement of a PEG tube. Patient 3 was an obese, poorly controlled diabetic and hypertensive who suffered multiple cerebrovascular events, indicative of endothelial dysfunction/chronic inflammation. These three patients are convincing examples of MICS associated with low PTH. One common theme among these three patients was that their PTH levels started trending down despite worsening EGFR once chronic inflammation and malnutrition started setting in.

In addition to the similarities in the three patient profiles, there were a few noteworthy differences. First of all, each patient's age was significantly different, ranging from 47 to 81 years. Additionally, in patient 1, the corrected calcium was at the upper end of normal 10.2 mg/dL (8.3-10.4 mg/dL), suggesting the possibility of hypercalcemia as the underlying cause of low PTH. However, follow-up laboratory workup revealed normal ionized calcium hence ruling out hypercalcemia contributing to suppressed PTH. Compared to patients 1 and 3, patient 2 had a relatively rapid decline in overall health over a short course of six months. Patient 3 had mildly elevated corrected calcium levels 10.9 mg/dL (8.3-10.4 mg/dL). However, the clinical picture was not consistent with primary hyperparathyroidism or other etiologies of hypercalcemia. Furthermore, with a mild elevation in calcium levels, PTH should not be suppressed to this extent. Moreover, suppressed PTH persisted despite lack of vitamin D supplementation, again suggesting MICS to be the underlying etiology to low PTH.

It is highly unlikely that any of our three patients could have ABD. Although ABD is clinically diagnosed based on low PTH and alkaline phosphatase, the gold standard for diagnosis of ABD is by histomorphometric analysis of tetracycline double-labeled bone biopsies [20]. However, difficulty in obtaining bone biopsies has made the procedure obsolete.

It is noteworthy that patients with advanced CKD and ESRD have a high prevalence of chronic inflammation and malnutrition and have a high prevalence of MICS. However, it is unclear as to why only some patients are found to have low PTH. Therefore, more research may be needed to look into the correlation between MICS and low PTH. Also, patients with low PTH due to MICS may miss out on the benefits of vitamin D analogs and calcimimetics supplementation, which is vital to maintaining bone health in patients with advanced CKD/ESRD.

## Conclusions

Low PTH levels seen in some CKD and ESRD patients may not necessarily be due to ABD but could also be a manifestation of the MICS. Instead of withholding treatment with active vitamin D analogs or calcimimetics, it is unclear if interventions to improve malnutrition and chronic inflammation will help address hypoparathyroidism and presumed ABD in patients with CKD and MICS. Clinical trials evaluating MICS, its etiologies, and effects would help guide the nephrology community on improving clinical outcomes in patients with ESRD.
